# Development of new anticoagulant highly honoured: Bayer’s Xarelto® recognised with 2010 international Prix Galien award

**Published:** 2010-12

The highly distinguished Awards Committee of the Galien Foundation has honoured Bayer’s Xarelto® (rivaroxaban) with the Prix Galien International 2010 in the category Best Pharmaceutical Agent. Xarelto® had previously already been recognised with national Prix Galien awards in Belgium, France and Switzerland.

The Prix Galien award recognises outstanding achievements in improving health through the development of innovative therapies, and is regarded as the equivalent of the Nobel Prize in biopharmaceutical research. The Awards Committee of the Prix Galien International 2010, including many Nobel Prize laureates, was chaired by Gerald Weissmann, MD, professor of Rheumatology and director of Biotechnology Study Centre, New York University School of Medicine. The award ceremony was hosted at the American Museum of Natural History in New York, USA.

‘We are very excited about the award and honoured by the recognition of this globally renowned committee. Being awarded the International Prix Galien for Best Pharmaceutical Agent underscores the drive for innovation that characterises the focus of our company, and our continuing ambition to improve the quality of life of patients’, commented Dr Marijn Dekkers, chairman of the Board of Management of Bayer AG. ‘Xarelto® has consistently demonstrated superior efficacy compared to current standard therapy for the prevention of venous thromboembolism in patients undergoing total hip- or knee-replacement surgeryand has become the market leader amongthe new oral anticoagulants.’

## About rivaroxaban

Rivaroxaban is a novel oral anticoagulant that was invented in Bayer Schering Pharma’s Wuppertal laboratories in Germany, and is being jointly developed by Bayer HealthCare and Johnson & Johnson Pharmaceutical Research & Development, LLC. In clinical studies, rivaroxaban has consistently shown superior efficacy to enoxaparin in preventing venous thromboembolism (VTE) in adult patients following elective hip- or kneereplacement surgery. It has a rapid onset of action with a predictable dose response and high bioavailability, no requirement for coagulation monitoring, and a limited potential for food and drug interactions.

Rivaroxaban is marketed under the brand name Xarelto® for VTE prevention in adult patients following elective hip- or knee-replacement surgery, and it is the only new oral anticoagulant that has consistently demonstrated superior efficacy over enoxaparin for this indication. Xarelto® is approved in more than 100 countries worldwide and has been successfully launched in more than 75 countries by Bayer Schering Pharma, achieving the market leader position among the new oral anticoagulants.

The extensive clinical trial programme supporting rivaroxaban makes it the most studied oral, direct factor Xa inhibitor in the world today. More than 65 000 patients are involved in the rivaroxaban clinical development programme, which is evaluating the product in the prevention and treatment of a broad range of acute and chronic blood-clotting disorders, including stroke prevention in patients with atrial fibrillation, secondary prevention of acute coronary syndrome, and VTE prevention in hospitalised medically ill patients.

